# The Online Patient Satisfaction Index for Patients With Low Back Pain: Development, Reliability, and Validation Study

**DOI:** 10.2196/21462

**Published:** 2021-11-15

**Authors:** Tamana Afzali, Henrik Hein Lauridsen, Janus Laust Thomsen, Jan Hartvigsen, Martin Bach Jensen, Allan Riis

**Affiliations:** 1 Research Unit for General Practice in Aalborg Department of Clinical Medicine Aalborg University Aalborg Denmark; 2 Departments of Sports Science and Clinical Biomechanics Centre for Muscle and Joint Health University of Southern Denmark Odense Denmark; 3 Nordic Institute of Chiropractic and Clinical Biomechanics Odense Denmark; 4 Department of Physiotherapy University College Northern Denmark Aalborg Denmark

**Keywords:** data accuracy, patient satisfaction, rehabilitation, low back pain, internet-based intervention, mobile phone

## Abstract

**Background:**

Low back pain is highly prevalent, and most often, a specific causative factor cannot be identified. Therefore, for most patients, their low back pain is labeled as nonspecific. Patient education and information are recommended for all these patients. The internet is an accessible source of medical information on low back pain. Approximately 50% of patients with low back pain search the internet for health and medical advice. Patient satisfaction with education and information is important in relation to patients’ levels of inclination to use web-based information and their trust in the information they find. Although patients who are satisfied with the information they retrieve use the internet as a supplementary source of information, dissatisfied patients tend to avoid using the internet. Consumers’ loyalty to a product is often applied to evaluate their satisfaction. Consumers have been shown to be good ambassadors for a service when they are willing to recommend the service to a friend or colleague. When consumers are willing to recommend a service to a friend or colleague, they are also likely to be future users of the service. To the best of our knowledge, no multi-item instrument exists to specifically evaluate satisfaction with information delivered on the web for people with low back pain.

**Objective:**

This study aims to report on the development, reliability testing, and construct validity testing of the Online Patient Satisfaction Index to measure patients’ satisfaction with web-based information for low back pain.

**Methods:**

This is a cross-sectional validation study of the Online Patient Satisfaction Index. The index was developed with experts and assessed for face validity. It was subsequently administered to 150 adults with nonspecific low back pain. Of these, 46% (70/150) were randomly assigned to participate in a reliability test using an intraclass correlation coefficient of agreement. Construct validity was evaluated by hypothesis testing based on a web app (MyBack) and Wikipedia on low back pain.

**Results:**

The index includes 8 items. The median score (range 0-24) based on the MyBack website was 20 (IQR 18-22), and the median score for Wikipedia was 12 (IQR 8-15). The entire score range was used. Overall, 53 participants completed a retest, of which 39 (74%) were stable in their satisfaction with the home page and were included in the analysis for reliability. Intraclass correlation coefficient of agreement was estimated to be 0.82 (95% CI 0.68-0.90). Two hypothesized correlations for construct validity were confirmed through an analysis using complete data.

**Conclusions:**

The index had good face validity, excellent reliability, and good construct validity and can be used to measure satisfaction with the provision of web-based information regarding nonspecific low back pain among people willing to access the internet to obtain health information.

**Trial Registration:**

ClinicalTrials.gov NCT03449004; https://clinicaltrials.gov/ct2/show/NCT03449004

## Introduction

### Background

Low back pain (LBP) is highly prevalent and is the most frequent reason for patients to consult general practice in Denmark [1,2]. LBP affects men and women of all ages [3] and is rarely caused by one specific factor [4,5]. Therefore, for most patients, their LBP is labeled as nonspecific, that is, a nociceptive source is not well established, and causes are multifactorial [6,7]. Patient education and information are generally recommended for people seeking care for nonspecific LBP [8]. However, delivering evidence-based information can be time-consuming and cumbersome, which can be a challenge during the available consultation time in general practice [9].

The internet is an accessible source of medical information for patients and it offers a range of information provided by a variety of sources. It has been reported that approximately 50% of patients search the internet for health and medical advice [10], and evidence suggests that this is increasing [11]. The advancement of new technologies offers more opportunities for delivering patient information on private computers, tablets, and smartphones, and web-based information can be considered an inexpensive solution to extend the treatment in general practice [12]. Therefore, future optimization of web-based information delivery has the potential to increase the delivery of evidence-based information about LBP, which may, in turn, lead to better patient outcomes [13,14].

Patient satisfaction is important in the use of web-based information and the degree to which patients rely on information from the internet [15]. Although patients who are satisfied with the information they retrieve use the internet as a supplementary source of information, dissatisfied patients tend to avoid using the internet [11]. Consumers’ loyalty to a product is often applied to evaluate their satisfaction [16]. Consumers have been shown to be good ambassadors for a service or product when they are willing to recommend the service to a friend or colleague [16,17]. When consumers are willing to recommend a service to a friend or colleague, they are also likely to be future users of the service [16,17].

### Objectives

To the best of our knowledge, no multi-item instrument to specifically evaluate satisfaction with information delivered on the web for people with LBP exists. This study aims to report on the development and validation of the Online Patient Satisfaction Index (OPSI), a self-reported measure to evaluate patients’ satisfaction with web-based information for LBP.

## Methods

### Overview

This study was registered at ClinicalTrials.gov (ID: NCT03449004). The study follows the Consensus-based Standards for the Selection of Health Measurement Instruments Taxonomy [18]. The *Methods* section consists of 2 subsections: development of the OPSI and validation of the OPSI.

### Development of the OPSI

#### Previous Work

A qualitative interview study had previously identified patients’ preferences for the content, design, and functionality of a web application with evidence-based information and advice for patients with LBP consulting general practice [19]. This study identified a set of important domains to address web-based information and advice for patients with LBP in Denmark and highlighted the importance of the following domains: design, readability, customization, credibility, usability, and coping [19]. On the basis of these findings, 8 specific items related to these domains were identified as important for patient satisfaction with web-based information for LBP. Design and readability were represented with 2 items; the other domains were presented with 1 item each.

#### Development Process

**Figure 1:**
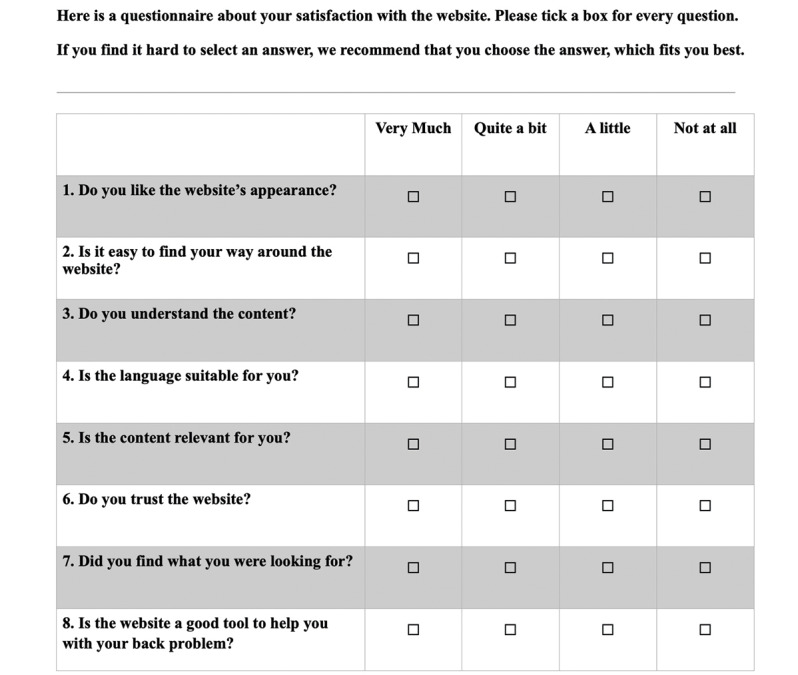
The Online Patient Satisfaction Index, translated to English from Danish.

A total of 8 items were combined into the first version of the index. The content of the 8 items in the first version came from an interview study [19], after which the authors made a draft version where experts (not members of the author group) and 10 patients provided input. Thereafter, the reliability and validity were tested. All items initially had response options ranging from 0 to 10, where 0 indicated strongly dissatisfied and 10 indicated strongly satisfied. The first version of the index was then tested for face validity by discussing the wording of the items with 7 experts. The experts were personally invited among colleagues but were outside the author group (1 academic and 1 researcher experienced in written communication, 2 researchers with expertise in musculoskeletal disorders, and 3 researchers with expertise within the development of questionnaires). This process was carried out through 2 rounds, where the first draft was discussed among the experts and subsequently modified. After round 1, the questions were reformulated to reduce jargon and for better wording of the items, and the order of the items was rearranged to create a better flow of the index. This revised index was then discussed with the same experts until a consensus on the final version was reached. Importantly, the 0 to 10 response rate scale about satisfaction was found to be difficult to use, and therefore, the response scale was changed from the numerical rating scale to a categorical scale about satisfaction with 4 response options: *Very Much, Quite a bit, A little,* and *Not at all* (Figure 1).

#### Conceptual Framework

The OPSI is based on a formative model in which the construct (satisfaction) is the result of patients’ experiences with different aspects of satisfaction. For example, the items relating to design, credibility, and readability can all have an impact on patients’ satisfaction with the web-based information, whereas higher satisfaction with a home page does not necessarily lead to patients finding it more customized to their needs [20].

#### Face Validity

**Figure 2:**
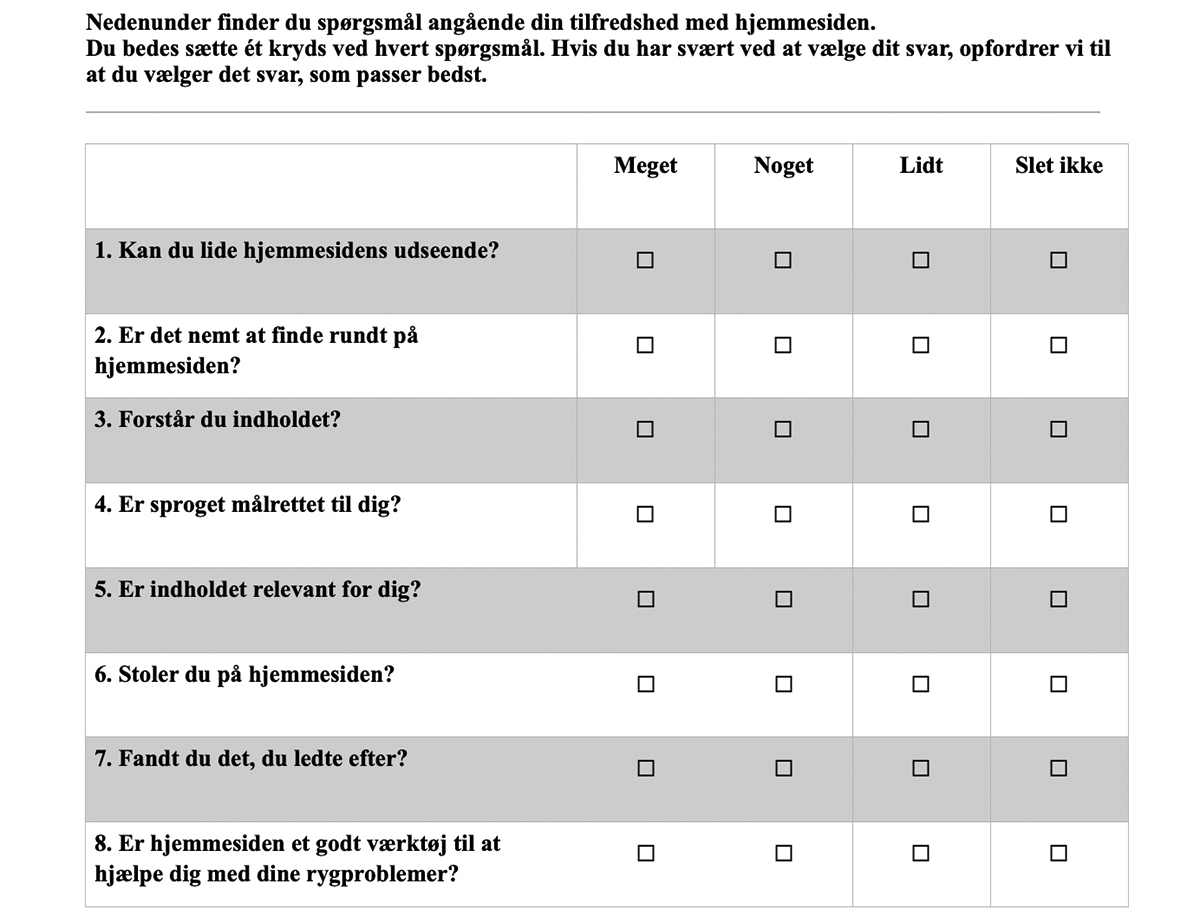
The Online Patient Satisfaction Index in Danish. Each item contributes 0 to 3 points, giving a total Online Patient Satisfaction Index score between 0 and 24.

The OPSI was pilot tested for face validity on 10 respondents with nonspecific LBP from the Sano Centre and 10 respondents with LBP recruited from social media. The Sano Centre is a training and rehabilitation center for people with a high degree of musculoskeletal pain or disability. First, they were asked to fill in baseline characteristics on paper for age, sex, pain duration (>12 weeks), pain intensity, curiosity to find new knowledge (0-10), and frequency of internet searching for health-related information (monthly or more). With 1 researcher (AR or TA) present, respondents were asked to search for information on an existing website (The Patient Handbook) [21]. The publicly available Patient Handbook has previously been found to be trustworthy and a preferred site among Danes searching for information about LBP [19]. The author group was not involved in developing the design or choosing the content of the Patient Handbook. After assessing the Patient Handbook for 10 minutes, respondents were asked to complete the OPSI and were encouraged to comment openly on the process and content. Their thoughts and comments were noted on paper by the researcher. This was done to optimize the content validity of the items by reducing ambiguity, avoiding double-barreled questions, reducing jargon terms, reducing the length of the items, checking the existence of irrelevant items, and patients were asked if there was a lack of any items related to patient satisfaction with web-based information. We specifically asked about the feasibility of the questions, their understanding of the items, and the reasons for their choice of response options. Respondents’ thoughts and suggestions about the index were discussed between TA and AR, and the index was revised and ready for validation among a larger population of respondents with nonspecific LBP. Figure 1 shows the English version of the OPSI, which has been forward-backward translated from Danish using the method suggested by Beaton et al [22] with modifications to stage 4. In stage 4, 1 native English-speaking researcher in expertise in musculoskeletal disorders achieved consensus with 2 native Danes holding a master’s degree in English. Although stages 1 to 3 were conducted at personal meetings, stage 4 was conducted on the web with TA as a facilitator. The Danish version is shown in Figure 2.

### Construct Validity and Reliability of the OPSI

Data from the validation study were gathered using paper versions of the OPSI, and TA entered the data in REDCap (Research Electronic Data Capture, Vanderbilt University) [23].

#### Participants

Sample sizes for validation studies are recommended to contain more than 100 participants and at least 50 participants per subgroup [20]. Consequently, the required sample size was set at 150 participants. The sample population for the development of the OPSI was recruited from Sano Centre in Aarhus, Denmark, and via social media (Facebook, LinkedIn, and Twitter) to obtain a case-mix. The patients had a 4-week stay at the center, and the training and rehabilitation course was patient-centered and tailored to the patients’ needs. Patients were offered specialized therapy, and most received an iPad as part of their training program [24]. Hence, most people were familiar with the internet and electronic devices to manage their pain. Respondents from social media were also expected to be familiar with the use of the internet and electronic devices. Consequently, the sample population was expected to be heterogeneous regarding their levels of pain and functional disability.

Respondents from Sano and social media were eligible for inclusion if they had nonspecific LBP (with or without leg pain) of any pain intensity during the previous year and were older than 18 years. Exclusion criteria were as follows: no internet access, pregnancy, inability to speak Danish as their native language, diagnosis of spinal stenosis, or signs of a serious underlying disease (signs of fracture, cauda equina syndrome, malignancy, osteoporosis, or spondyloarthritis).

#### Procedures

The study was registered by the Danish Data Protection Agency (J.nr. 2017-41-5222). Ethics approval was not required following Danish law. Respondents received verbal project information from TA or AR, and informed consent was signed by the respondents and the assessor. Testing was performed at the Sano Centre, public libraries, or in respondents’ homes with either TA or AR present. Initially, respondents filled in baseline questions on paper and were encouraged to navigate and search for a new home page, MyBack, for 10 minutes. The MyBack home page is in Danish and contains information about LBP to guide patients with self-management [25]. The content and design were developed by researchers with systematic input from patients and general practitioners [19,26,27]. Respondents then filled in paper versions of the OPSI to measure their satisfaction with MyBack together with other questions about satisfaction and their functional disability. If any item was left blank, the assessor encouraged respondents to choose the response that was most suitable for them. Thereafter, respondents navigated Wikipedia with information about LBP for 10 minutes with minimum help from the assessor. We assumed that most respondents would be more satisfied with MyBack than with information about LBP on Wikipedia. As the amount of information regarding LBP is limited in the Danish version of Wikipedia, we considered it difficult to read, not addressed to a particular group of people, and the sources are unknown and might, therefore, not be considered trustworthy by respondents. Thereafter, respondents filled out the OPSI for the Wikipedia page.

Among the respondents, 70 were randomized to be invited to participate in the retest after a minimum of 7 days and a maximum of 4 weeks. Randomization and allocation numbers were provided by a researcher who was not involved in this study. A publicly available home page was used to generate 150 numbers with *yes* or *no* and sent the document with the allocation numbers to the assessors [28]. Respondents randomized to *yes* were invited to participate in a retest. Respondents who agreed to take part in the retest searched and navigated the MyBack home page a second time after a minimum of 7 days with the same assessor present (TA or AR). After filling out paper versions of the OPSI, respondents were encouraged to respond to 1 question about the stability of their satisfaction: Do you think your satisfaction with the home page has changed since last time? (Answer options: yes/no/don’t know). Their replies were discussed with the assessor to validate their responses and if responding *no* or *don’t know*, they were considered stable and included in the retest analysis.

#### Measurement Tools

A web app can be considered a product, and we assume that users are satisfied when they are more likely to recommend a web app to a friend or colleague. *The Ultimate Question* was used as the primary outcome measurement to compare measurement properties with the OPSI. *The Ultimate Question* is often applied to measure costumers’ satisfaction with products or services using a single question: How likely are you to recommend the website to others? The question can be answered using a 0 to 10 scale, where 0 is the least likely to recommend and 10 is the most likely to recommend [16,17]. Respondents replying 10 or 9 are considered promotors and are likely to buy or use the product or service again, respondents replying 8 or 7 are considered passive, and respondents replying 6-0 are considered detractors [16,17]. Subtracting the percentage of detractors from the percentage of promoters yields the net promoter score (NPS).

For this study, we dichotomized the scores by defining the responses 10 or 9 as *promoters* of the web-based information and, therefore, considered satisfied, whereas we defined responses from 8 to 0 as *nonpromotors* and thereby not satisfied [16,17]. A Global Rating Scale (GRS) of satisfaction with the web app was applied (*What is your overall satisfaction with the website?)* with response options ranging from 10 (very satisfied) to 0 (not at all satisfied).

The Danish version of the Roland-Morris Disability Questionnaire (RMDQ) was used to measure LBP related function [29]. The Danish version consists of 23 items, and the sum score ranges from 0 (no disability) to 23 (maximum disability) [29].

#### Statistical Evaluation

Reliability was assessed by studying the difference between the OPSI at baseline and OPSI after a minimum of 7 days using a 2-way mixed-effect analysis of variance model with interaction for absolute agreement as the intraclass correlation coefficient of agreement (ICC_agreement_) [30]. Where an ICC_agreement_ >0.75 can be interpreted as excellent, 0.4 to 0.75 indicate fair to poor, and values <0.4 indicate poor reliability [31]. Measurement error was assessed using the limits of agreement proposed by Bland and Altman [32]. The smallest detectable change (SDC_consistency_) was calculated as follows:

SDC_consistency_ = 1.96 × SD_difference_

where SD_difference_ is the SD of the difference between the test and retest. This equals the limits of agreement without systematic errors [33]. Construct validity was evaluated by hypothesis testing of the size and direction of correlations between the OPSI score for MyBack and the NPS, GRS, RMDQ, and OPSI score for Wikipedia about LBP using Spearman rank correlation coefficient. CIs were estimated by bootstrapping with 5000 replications. Correlations between 0.3 and 0.5 were considered weak, and correlations >0.5 were considered strong [34].

#### Hypothesis 1

We hypothesized that being categorized as *promotors* (scoring 9 or 10 on the NPS) would be positively and strongly (>0.5) correlated with higher OPSI scores (convergent validity).

#### Hypothesis 2

We hypothesized that respondent scores from 9 to 10 on the GRS would be positively and strongly (>0.5) correlated with higher OPSI scores (convergent validity).

#### Hypothesis 3

We hypothesized that RMDQ scores would be positively and weakly (<0.3) correlated with higher OPSI scores (discriminant validity).

#### Hypothesis 4

We hypothesized that the OPSI score for MyBack and the OPSI score based on the Wikipedia website would be negatively and weakly (−0.3 to 0) correlated with higher OPSI scores for OPSI based on MyBack (discriminant validity).

All analyses were conducted using Stata 16.0 (Stata Corp).

## Results

A total of 150 participants were recruited between March 6, 2018, and May 10, 2019. The mean age of the participants was 48.7 (SD 12.9) years, and 67.3% (101/150) were women. Most (146/150, 97.3%) had experienced pain for >12 weeks with an average score of 8 (range, 0-10) for having an interest in finding new information on the internet (Table 1).

**Table 1:** Characteristics of the study population (N=150).

Baseline characteristics		Patients
Age (years), mean (SD)		48.7 (12.9)
Female, n (%)		101 (67.3)
Education level, bachelor’s degree or more, n (%)		55 (36.7)
**Employment status, n (%)**		
	Working full-time or part-time	66 (44)
	On sick leave or leave of absence	39 (26)
	Unemployed	8 (5.3)
	Retired	37 (24.7)
Pain duration >12 weeks, n (%)		146 (97.3)
Pain intensity, 0-10, mean (SD)		5.4 (2.1)
RMDQ^a^ score, mean (SD)		11.8 (5.1)
Contact with GP^b^ about LBP^c^ during the past 1 year, n (%)		146 (97.3)
Curious about finding new information, 0-10, median (IQR)		8 (7-9)
**Use of internet about health, n (%)**		
	Daily	9 (6)
	Weekly	48 (32)
	Less than weekly	10 (6.7)
	Monthly	39 (26)
	Less than monthly	44 (29.3)
OPSI^d^ home page, median (IQR)		20 (18-22)^e^
OPSI Wikipedia, median (IQR)		12 (8-15)^f^
OPSI home page retest, median (IQR)		20 (17-22)^g^

^a^RMDQ: Roland-Morris Disability Questionnaire.

^b^GP: general practitioner.

^c^LBP: low back pain.

^d^OPSI: Online Patient Satisfaction Index.

^e,f,g^Distribution of the OPSI scores were nonnormal, which were identified on histograms and q-norm visualizations.

**Figure 3:**
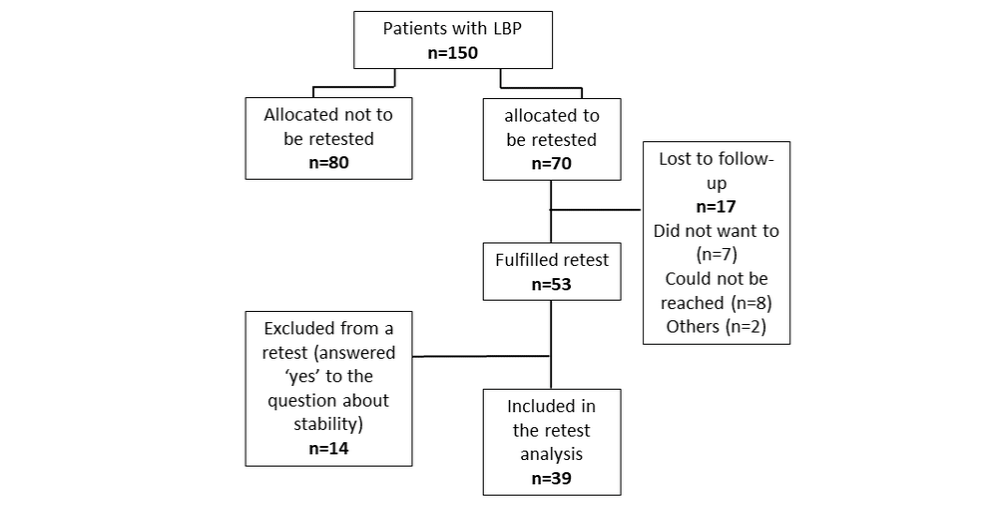
Flowchart of respondents. A total of 150 respondents were included in the validity analyses, and 39 were included in the reliability analysis. LBP: low back pain.

A total of 70 randomly chosen participants were invited to the retest, 53 accepted whereas 39 answered *no* or *don’t know* to the question about the stability of their satisfaction. These 39 were considered stable and were included in a retest analysis to evaluate the stability of the OPSI (Figure 3).

For the MyBack website, the OPSI score ranged from 724, and for the Wikipedia website, the entire OPSI score (0-24) range was used. The mean OPSI score of promoters for the MyBack website was 21.68 (95% CI 21.14-22.22), and the mean OPSI score of the nonpromoters was 18 (95% CI 17.25-18.75). The response rate was 100% for both websites. ICC_agreement_ was estimated at 0.82 (95% CI 0.68-0.90). The SDC_consistency_ was estimated at 4.71.

**Figure 4:**
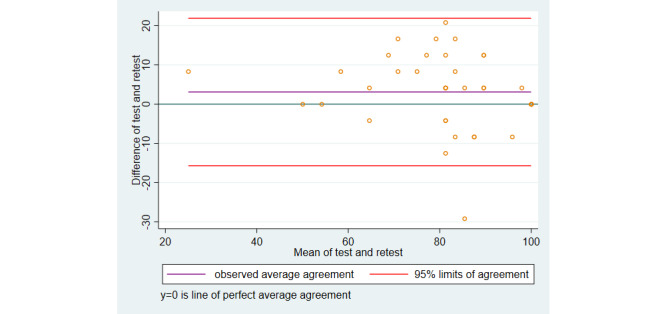
Comparison of test-scores and retest-scores. Online Patient Satisfaction Index for MyBack at the initial test and retested after 1 week.

Limits of agreement were estimated to be −4.11 to 5.13 with a mean difference of 0.509 (95% CI −0.127 to 1.146). The mean difference is close to zero with the CI overlapping zero; hence, there is a negligible systematic difference between the baseline measurement and the retest measurement. In addition, the difference between baseline and follow-up measurements did not seem to depend on the level of satisfaction (Figure 4).

Two hypotheses were confirmed (Table 2).

**Table 2:** Hypothesis testing of construct validity.

Characteristics	Hypotheses	Correlations^a^	
		Expected, *r*	Observed^b^, *r* (95% CI)
NPS^c^	Hypothesis A^d^	>0.5	0.58 (0.47 to 0.69)
GRS^e^	Hypothesis A	>0.5	0.70 (0.60 to 0.80)
RMDQ^f^	Hypothesis B^g^	<0.3	−0.14 (−0.30 to 0.02)
OPSI_wikipedia_^h^	Hypothesis C^i^	−0.3 to 0	−0.04 (−0.20 to 0.13)

^a^Spearman rank correlation coefficient.

^b^Correlations from confirmed hypotheses.

^c^NPS: net promoter score.

^d^Scales are expected to measure the same construct. The correlation was expected to be positive and strong (>0.5).

^e^GRS: Global Rating Scale.^f^RMDQ: Roland-Morris Disability Questionnaire.

^g^Scales are not expected to measure the same construct. The correlation was expected to be positive and weak (<0.3).

^h^OPSI: Online Patient Satisfaction Index.

^i^Scales are expected to measure the same construct. The correlation is expected to be negative and weak (−0.3 to 0).

## Discussion

### Principal Findings

The OPSI is easy to apply as each of the 8 items is scored from 0 to 3, resulting in an overall score between 0 and 24 points. The index was found to have good face validity, excellent reliability, and good construct validity among our sample of participants with long-standing nonspecific LBP.

### Recommendations to the OPSI Users

We recommend the use of proportional recalculation to convert the index score to a 0 to 100 scale, as it accounts for items with missing scores [35]. The OPSI was found to have excellent reliability in measuring satisfaction at 1 time point. Thus, it is suitable to measure satisfaction at 1 time point for 1 person or to compare satisfaction between groups. However, we did not test for responsiveness among participants experiencing a change in satisfaction over time; consequently, we cannot recommend using the OPSI to measure changes over time. Whether this can be recommended in the future, needs to be supported by the evaluation of responsiveness.

### Limitations

The item *Do you trust the website?* is expected to capture both trust in the content and trust in security and handling of data. During development and face validity, splitting this item into two was not mentioned by participants. This may be explained by the high level of trust in health care authorities handling data in Denmark, but we do not know if this is the case. In other cultures, trust in the content and trust in the handling of data by the provider may be considered as 2 different issues and thereby require 2 items to be properly captured.

The development and evaluation of the OPSI was based on a formative model, which is 1 of the 2 conceptual frameworks, the other being a reflective model. A reflective model assumes interrelatedness between items and thereby item correlations. However, a formative model does not assume item correlations, and this is a limitation, as common statistical methods to describe the relationships between items and the construct were not applicable in this study [20].

The Sano Centre receives a new cohort of patients every 4 weeks, most of whom have long-standing LBP, so it is convenient and easy to ask patients to participate in the study because they stay at the center. For participants volunteering over social media, the assessors had to make an appointment to meet at a convenient location, which was not always easy and straightforward. Consequently, this led to an unequal distribution of respondents between the 2 sites, with (131/150, 87.3%) from the Sano Centre and (19/150, 12.7%) from social media. This is a limitation of the study as patients from the Sano Centre were expected to have more severe symptoms, require extended information, and thus score a larger difference between the 2 home pages. In contrast, participants recruited from social media may be more frequent users of Wikipedia for other information seeking and thereby more satisfied with the shorter wiki format. The size and direction of the Spearman coefficient changed for patients recruited from social media. However, this could be due to the small number of patients recruited from social media.

The time between the test and retest in the analysis of reliability was between 1 and 2 weeks, with a maximum of 4 weeks, which might have overestimated the reliability of OPSI. The short time duration may have influenced the participants’ responses as some may recall their previous baseline response and, therefore, repeat the answer. However, people with back pain often experience changes in symptoms, and the short period between tests can be considered a strength when collecting data from participants with possible fluctuations in symptoms within a few weeks [36]. We applied a stability question to determine whether the participants were considered stable. It is a strength that TA or AR discussed with the participants to ensure that participants understood the question on change in satisfaction regarding MyBack and to ensure that participants with a change were excluded from the retest. We used the construct of *satisfaction* as a reflection of whether the included respondents in the reliability study were stable, and this is in fact treating the items as a reflective model. However, the alternatives would either be to ask the respondents if they had changed each item or not to ask them at all. The first alternative would cause analytic problems determining who were stable and who were not, as some respondents probably would have changed on 1 or perhaps 2 items but not the rest. Setting a cutoff point on an acceptable number of stable items would be arbitrary and probably misleading, and we also question the feasibility of doing it this way. The second alternative of not asking about s*tability* is, in our opinion, unacceptable, as the potential to introduce a bias is high. We therefore opted for the solution of implementing a *global change question* as we believe this is the best of the 3 options introducing least bias regarding choosing stable respondents. However, assuming *don’t know* as stable is potentially a limitation.

The use of NPS as a comparator for the construct validity of OPSI to measure satisfaction might be a weakness. The NPS uses a proxy to assess the customer’s overall satisfaction with a service or product [17]. NPS is based on only 1 question with a reply option from 0 to 10, which is categorized into 3 groups: promoters (10-9), passive (8-7), and detractors (6-0) [17]. When estimating satisfaction, the middle group (passive) was excluded from the analysis [17]. This is, in our opinion, a weakness that can limit the usability of the NPS as a comparator for construct validity. We also applied the GRS as a comparator for construct validity using a response scale ranging from 0 to 10. The GRS may be inflated due to recall bias [37] and motivational effects [38] in longitudinal studies. Furthermore, transition scores seldom show an ideal pattern of association between baseline and follow-up measures [37]. However, we used baseline GRS to compare with a baseline score for the OPSI, and using a transition score to compare baseline scores has previously been found feasible [37].

### Comparison With Prior Work

Although we have found no previous studies evaluating satisfaction with web-based information on LBP, other studies have reported satisfaction with web-based delivered health care information and educational information. Hence, a recent study of satisfaction with a gamified medical course among medical students in Thailand found a mean satisfaction of 9.02 (SD 1.11) out of 10 [39]. Equal high satisfaction was recorded in a study about the satisfaction of SMS text containing educational material for patients undergoing prostate biopsy in the United States. This study found a mean satisfaction of 4.5 (SD 0.9) out of 5 [40]. Furthermore, a high level of satisfaction was found in a study of satisfaction with telephone support in patients with type 2 diabetes [41,42]. They recorded a mean of 4/5 on all 21 items related to satisfaction [42]. These previous studies indicate a problem with ceiling effects when measuring satisfaction with health care interventions. The median score of OPSI used on a home page, which we considered good, was 20 [18,22] out of a maximum of 24 points. This indicates that satisfied patients tended to reply at the high end. Furthermore, this is in line with the development and use of the NPS, where only customers scoring 9 or 10 on a scale from 0 to 10 are considered satisfied with a product or a service to a degree where customers are likely to buy a product again or reuse the service [16].

The reliability test indicated that a change of 4.71 points is necessary to preclude measurement error. If performing proportional rescaling of OPSI to a scale from 0 to 100, a change of approximately 20 points is necessary to preclude measurement error. In another study, applying a 0 to 100 score, the minimal detectable change was found to be 32.8 points on the group level when measuring the individualized quality of life in patients with LBP [43]. In a reliability and responsiveness study evaluating the minimal detectable change in LBP disability, questionnaires found lower or similar minimal detectable changes of 8.6 (RMDQ), 15 (Oswestry Disability Questionnaire), and 22 (the 36-item Short Form health surveys physical functioning scale) [44]. Consequently, compared with other measures applied to patients with LBP, OPSI has a similar good reproducibility.

### Unanswered Questions and Future Research

The OPSI was evaluated in a population with severe and disabling LBP. Consequently, the index will benefit from further validation in other populations with less severe symptoms. In addition, the OPSI can be tested as a more generic tool to assess satisfaction without including dissatisfaction. King et al [45] constructed a survey with 22 items about the usability of phone apps to support physical activity and rated it on a 6-point Likert-type scale [45]. This scale was later adapted into a 21-item questionnaire rated for agreement or disagreement on a 5-point Likert scale [46]. A questionnaire developed more recently to measure user satisfaction with mobile health (mHealth) apps also applied a Likert scale—from *strongly disagree* to *strongly agree* [47]. However, this questionnaire did not meet the criteria for unidimensionality [47]. The OPSI is not challenged by the same potential limitation, as only satisfaction is measured and dissatisfaction is not.

We did not collect specific data regarding health literacy; however, either volunteering to participate on the internet or participating in a 4-week course at Sano indicates a health-interest in seeking health information among participants. This interest is supported by participants’ baseline characteristics regarding the use of the internet to search for health information. Engaging all people, particularly those with low health literacy, in assessing health information can be a challenge [48]. Nevertheless, future research is needed to evaluate whether the OPSI can also be used by people with low health literacy. The index was developed and formally evaluated in Danish; however, it was translated into English using established guidelines [22]. The OPSI is potentially applicable in all Western countries, but future studies need to evaluate validity and reliability among other cultures and in other languages.

### Conclusions

The OPSI showed good face validity, excellent reliability, and good construct validity and can be used when measuring satisfaction with the provision of information regarding LBP among people willing to access the internet for health information.

## Abbreviations

GRS: Global Rating Scale
ICC: intraclass correlation coefficient
LBP: low back pain
NPS: net promotor score
OPSI: Online Patient Satisfaction Index
REDCap: Research Electronic Data Capture
RMDQ: Roland-Morris Disability Questionnaire
SDCconsistency: smallest detectable change
